# Effects of Medium-Term Amendment with Diversely Processed Sewage Sludge on Soil Humification—Mineralization Processes and on Cu, Pb, Ni, and Zn Bioavailability

**DOI:** 10.3390/plants7010016

**Published:** 2018-03-02

**Authors:** Gabriella Rossi, Claudio Beni

**Affiliations:** Consiglio per la Ricerca in Agricoltura e L’analisi Dell’economia Agraria, Centro di Ricerca Agricoltura e Ambiente (CREA-AA), via della Navicella 4, 00184 Roma, Italy; claudio.beni@crea.gov.it

**Keywords:** sewage sludge amendment, soil organic carbon, composted sewage sludge, heavy metals bioavailability

## Abstract

The organic fraction of sewage sludge administered to agricultural soil can contribute to slowing down the loss of soil’s organic carbon and, in some cases, can improve the physical and mechanical properties of the soil. One of the main constraints to the agricultural use of sewage sludge is its heavy metals content. In the long term, agricultural administration of sewage sludge to soil could enhance the concentration of soil heavy metals (as total and bioavailable fractions). The aim of this research was to evaluate the effects of medium-term fertilization with diversely processed sewage sludge on the soil’s organic carbon content and humification–mineralization processes, on the physical–mechanical properties of soil and their influence on the pool of potentially bioavailable heavy metals, in order to assess their effectiveness as soil organic amendments. After eight years of sludge administration; an increase in the concentrations of bioavailable form was evidenced in all the heavy metals analyzed; independently of the type of sludge administered. The form of sludge administration (liquid, dehydrated, composted) has differently influenced the soil humification–mineralization processes and the physical–mechanical properties of soil. The prolonged amendment with composted sewage sludge contributed to keeping the soil humification–mineralization process in equilibrium and to improving the physical and mechanical qualities of the treated soil.

## 1. Introduction

Soil organic matter influences and regulates the cycle of the nutrients in soil–plant systems, contributing significantly to the maintenance of global soil fertility (chemical, physical, and biological). It is involved, in fact, both in plant and micro-organism growth and, as a substrate for the mineralization processes, in the promotion of the soil’s nutrient availability. Therefore, the quantity, quality and turnover of the organic matter (OM) are correlated to potential fertility in natural and agricultural soils.

Mediterranean agricultural areas are characterized by low soil organic carbon (SOC) contents and are often prone to soil degradation and SOC depletion due to the changes in extensive land use in recent decades and they are highly vulnerable to environmental changes [1,2,3].

Continuously high temperatures during the summer in the Mediterranean lead to a rapid decline in the organic matter (OM) content in cultivated soil. The loss of the soil’s organic matter from agricultural soil is increasing very rapidly because of the climatic characteristics and the intensive land use which affect the processes of humification–mineralization of native soil organic matter.

A greater effort has to be given to conservation and enrichment of the organic matter in arable lands. Nowadays, growing concern focuses on the suitability of bio-wastes such as sewage sludge, municipal solid waste (MSW), and animal slurries to be used in agriculture. The promotion of waste recycling and recovery techniques is an urgent priority. The necessity of linking agricultural practices to sustainable environment management leads to the identification of farm procedures with beneficial effects on the ecosystem. Fertilization practices with organic waste could have a positive influence on the soil’s fertility and the soil’s nutrient supply [4,5]. Residual biomasses—such as sewage sludge, organic waste, and food industry residue—can enrich soil with macronutrients such as nitrogen, phosphorus, potassium, sulphur, calcium, magnesium, and micronutrients [6,7,8] which meet the requirements of cultivated crops and enhance the restoration of soil fertility. Approximately 50% of the solid fraction of sewage sludge is organic matter, which has a significant effect on the physical, chemical, and biological properties of the soil as a result of its application. Organic matter improves soil porosity and increases water retention and movement. Some components of organic matter play an important role in soil aggregation. 

Sewage sludge is a potential source of nutrients for crops, but its heavy metal content is a hazard to the environment. While few heavy metals are essential for living organisms in trace quantities, most are hazardous in high concentrations. Nowadays, the environmental directives in many countries regulate the use of sewage sludge in agriculture by limiting the total heavy metal concentration in sludge and soil; in Italy, the use of sludge in agriculture is regulated by Decree-law 99/92 [9] in implementing Directive 86/278/EC. In order to assess the potential for the re-use of sewage sludge in agriculture, its contribution to soil organic processes should be verified [10,11,12]. In addition, the effects on the physical and mechanical characteristics of the soil [13,14,15], on the behavior of heavy metals in the soil and in the soil–plant system and the hazard of environmental impact [16,17] should be evaluated. The bioavailable heavy metal fraction, which is potentially assimilated by biota or liscivable, is of great interest in soil contamination studies because it is the environmentally most mobile [18,19].

The aim of this research was to evaluate the effects of medium-term fertilization with diversely processed sewage sludge on the soil’s organic carbon content and humification–mineralization processes, on the physical–mechanical properties of soil and their influence on the pool of potentially bioavailable metals in order to assess their effectiveness as soil organic amendments.

## 2. Results 

### 2.1. Soil Organic Carbon Behavior in Amended Plots

Soil organic carbon (SOC), total extractable carbon (TEC) and humified carbon contents (HA + FA) were higher in amended plots than in control T (Table 1). In soil treated with sewage sludge, soil organic carbon increased between 26% (L; C) and 28% (D) in respect to T; the TEC increased between 17% (L) and 57% (C) with an intermediate value of 39% in D; humic and fulvic acid carbon increased between 24% (L) and 54% (C). No significant differences in SOC and TEC were found between amended plots. Only in plots treated with composted sewage sludge (C) were the concentrations of TEC and humic and fulvic acids carbon (HA + FA) significantly higher in comparison with L and D. 

**Figure 1:**
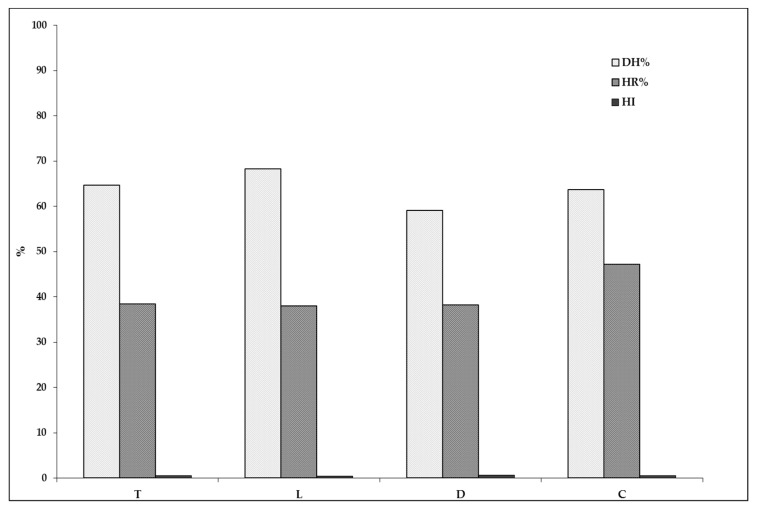
DH%, humification degree; HR%, humification rate; HI, humification index.

In Figure 1, the humification parameters are shown: DH (degree of humification), HR (humification rate), and HI (humification index) [20,21]. Humification parameters are commonly considered as an index of soil humification activity as well as of availability of non-humified labile fractions. 

In soil treated with composted sludge (C), DH% and HI showed values (DH% = 63.7; HI = 0.57) close to the control T (DH% = 64.7; HI = 0.54). This trend indicates that the addition of composted sludge has not altered the natural process of soil humification; the latter is favored by the higher concentration of humified carbon (HA + FA) (Table 1) and is also evidenced by the highest value of humification rate (HR% = 47.2). The liquid sludge (L) administration also contributes to keeping the humic fraction stable (HI = 0.46; DH% = 68.3). In contrast, dehydrated sludge (D) did not contribute to the humification processes. These treatments gave the highest value of humification index (0.69). The result, due to an increase of the not-humic extracted carbon in comparison with the humified carbon, indicates a drop in the humification level. It is strengthened by the lowest DH% value observed in this treatment (59.1).

**Figure 2:**
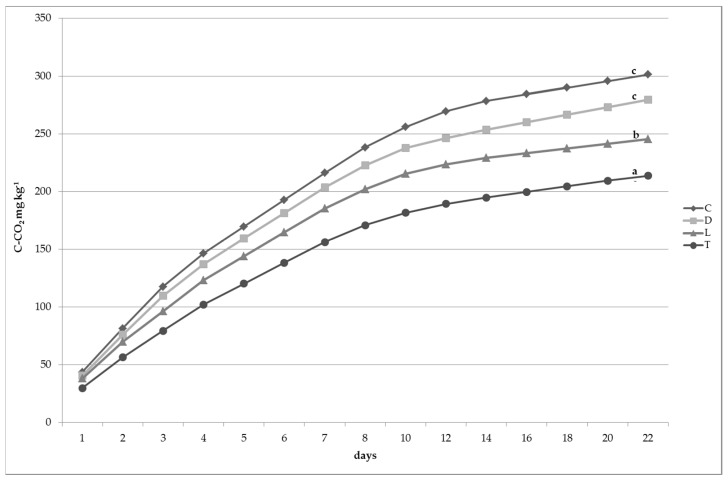
Cumulative CO_2_-C evolution from soil (mg kg^−1^), values marked with the same letter do not differ significantly at *p* ≤ 0.05 (LSD test), *n* = 12.

The cumulative amounts of CO_2_-C evolved from control (T) and from amended plots were determined to evaluate the soil carbon mineralization process (Figure 2).

The application of the amendments to soil induced a remarkable effect on the cumulative mineralized carbon. In amended plots the total amount of CO_2_-C mineralized increased significantly in respect to soil not treated (T). The CO_2_-C curves of differently treated soil indicated that plots amended by composted and dehydrated sludge gave the highest values of mineralized carbon. In particular, respiration rates were higher in C and D than in control (T), 41% and 31% respectively. This trend can be explained by analyzing the not humified and more labile carbon fraction (NHC). Samples of soil treated with dehydrated (D) or composted sludge (C) contained a greater quantity of NHC, in comparison with T and L. Indeed, NHC values, were 2.9 g kg^−1^ for D and C in respect to 1.8 g kg^−1^ (T) and 1.9 g kg^−1^ (L).

### 2.2. Effects of Sewage Sludge on the Physical and Mechanical Parameters of the Soil

All the physical–mechanical parameters have been improved following the use of composted sewage sludge compared to other treatments. In fact, in C treatment the soil deformation was lower at both 10 daN and 200 daN showing a higher bearing capacity of the soil, while shear strength and penetration resistance were lower, demonstrating the greater soil workability. The maximum hydraulic capacity was higher in C compared to other plots (Table 2).

### 2.3. Heavy Metal Bioavailability

With regard to total heavy metals (Table 3), Cu and Pb did not differ from control (T) in any treatment. Instead Ni and Zn showed a quite different behavior. The Ni soil concentration increased significantly in L, D, and C compared to control (T). However, no differences were observed among composted, liquid, and dehydrated treated plots. Zn content was significantly higher in D and C than in T and L.

With regard to bioavailable forms (Table 4), Cu contents were significantly higher in D and C in respect to T and L, while Pb concentration was significantly lower in L compared to other treatments. For Ni and Zn there was an overall increase in respect to control (T).

For the metals analyzed, the percentage of bioavailable fraction on the total concentration was calculated (DTPA-extractable/Total × 100). The percentages of bioavailability of copper and lead did not show significant variations between the control and the theses treated, reaching around 18% and 20% for copper and 10% and 12% for lead in respect to total forms. Zinc and nickel showed different behavior. For Ni, the percentage of bioavailable form in respect to total has varied from 1.15% (T) to 2.62% in D and 2.18% in C; in the case of Zn the percentage of bioavailability was 2.65% in T, 5.33% in L, 5.65% in D, and 4.31% in C.

## 3. Discussion

After eight years of administration to the soil, the use of sewage sludge as soil organic amendment has contributed to increasing the soil’s organic carbon content. The annual addition to the soil of the organic fraction of sewage sludge has contributed to improving not only the total soil organic carbon content but also the extractable and humified soil organic fractions, depending on the processing techniques. The analysis of humification parameters and soil respiration activity showed that the amendment with composted sewage sludge contributed positively, more than the other treatments, to the humification process. This result is comparable to what was found by Fernadez et al. [22] which showed that the organic matter of a composted sewage sludge is more stable than that of a dehydrated sludge. Moreover, regarding the present study, the easily decomposable carbon added to the soil by the composted sludge has favored the process of mineralization of the organic substance.

In amended soil, tillage is a fundamental factor in influencing soil quality, plant behavior, and the sustainability of farming systems [12] because it can alter the soil’s physical properties and the soil’s profile depth. Many authors use the soil penetration resistance measurements as a tool for characterizing soil strength after tillage [13]. The deformation response of a soil under different load conditions is used to predict how the soil stability changes after the application of an effective stress. Soil moisture content depends also on the organic matter content, influenced by the amendment. Soil moisture content during tillage affects the distribution of aggregates’ size and those aggregates formed at low moisture content have three to four times more resistance to crushing than those formed at greater moisture contents [14]. Consequently, an amendment allowing the increase of the soil’s organic matter also increases the moisture content of the soil; in these conditions, the soil aggregates are characterized by greater stability and the soil stability and workability indexes improve. The results obtained by the sewage sludge administration on the physical and mechanical parameters of soil showed an improvement of the soil tillage indexes in the soil treated with composted sewage sludge in comparison with other treatments.

An increase in concentrations of total nickel and zinc was detected in the soil. For bioavailable form (DTPA-extractable) this trend was evidenced in all heavy metals analyzed, independently of the type of sludge administered. These results suggest that long-term soil amendment with sewage sludge might shift the soil’s total heavy metals forms to bioavailable forms that are potentially more mobile, labile, and available to soil organisms and plants. In fact, the soil’s inorganic and organic components may form different kinds of bonds with micronutrients and heavy metals, influencing their bioavailability [23,24,25]. For instance, Zn^2+^ and Ni^2+^ show a tendency to form organic complexes with amide and amino ligands thus increasing their mobility in soils with respect to Cu and Pb which tend to be sorbed by cation exchange, especially by humic and fulvic acids [26,27].

In any of the cases, the amendment with sewage sludge diversely processed did not influence the balance between the total and bioavailable form of the heavy metals considered. In the case of copper and lead, potentially available forms are distributed in the same percentages in both untreated and treated soils, despite the yearly administration of the two metals contained in sewage sludge. In the case of zinc and nickel, the percentage of bioavailable form is considerably increased in the theses treated in respect to the control (T) but with similar values among the amended theses (L, D, C). 

This study highlighted that, comparing the results obtained with diversely processed sludge, the added organic fraction to soil by the prolonged amendment with composted sewage sludge contributed to keeping the soil humification–mineralization process in equilibrium and to improving the physical and mechanical qualities of the treated soil. Therefore, the use of composted sewage sludge would be more effective with respect to liquid or dehydrated sludge mainly in agricultural areas subject to the risk of depletion of the organic carbon of the soil with a consequent decrease in the soil’s fertility. However, it would be advisable to monitor the heavy metal concentrations in soil to verify that they always remain below legal limits when the sludge amendment practices are carried out for a long time.

## 4. Materials and Methods

Field trials were settled as a long term experiment with the aim of monitoring the benefits and drawbacks of sewage sludge administration to soil. 

### 4.1. Sewage Sludge

Sewage sludge was obtained from the municipal (5%) and agro-industrial (95%) waste treatment plant in Italy near Rome. Anaerobically digested dewatered (D, 24% d.w.), liquid (L, 3% d.w.) and composted (C, 62% d.m. 9:1 w:w sludge to straw ‘before-composting’ ratio) sludges were applied every year at a rate of 0 (T: control soil not treated) and 15 t ha^−1^ d.w. (L, D, C). The sludge used has been analyzed annually and its average characteristics and limits of the Italian law are reported in Table 5. The concentration of heavy metals in the sludge did not exceed the limits imposed by the D.L. 99/92 (Italian law).

Sludge was incorporated into the 0–30 cm plough layer every autumn. The experiment was carried out following a completely randomized block design. Plots of 7 × 7 m were laid out in four blocks and each treatment had a single replicate per block.

### 4.2. Soil Analysis

The soil was analyzed at the beginning of the study, before the sludge amendment, according to the official methods of the Ministry of Agriculture (Italy) [28]. Soil pH was determined with a glass electrode in a 2.5:1 water to soil ratio (v/w), as to particle size by the sedimentation procedure, cation exchange capacity (C.E.C.) by ammonium acetate procedure, total nitrogen by the Kjeldahl method [29] and available P with a spectrophotometer using the Olsen method [30].

The soil was characterized by a middle cation exchange capacity (C.E.C.), subalcaline pH, middle nitrogen content, low content of total organic carbon, and a low value of the C/N ratio (Table 6). The total concentrations of copper, lead, zinc, and nickel in the soil were below the limits set by Italian law.

After eight years, three sub-samples of soil (0–30 cm) for each replicate for each treatment were collected. Samples were air-dried at ambient temperature and the analyses were made on the <2 mm dried soil fraction after sieving. 

Soil organic carbon (SOC) was determined with the Springer and Klee [31], total extractable carbon (TEC), and humic and fulvic acid carbon (HA + FA) were determined by the dichromate oxidation method [32]. Not humified and more labile C fraction (NHC) was calculated by the difference [TEC − (HA + FA)]. Humification parameters DH (degree of humification), HR (humification rate), and HI (humification index) were determined according to Sequi et al. [20] and Ciavatta et al. [21]. DH% is given by (HA + FA × 100)/TEC, HR% by (HA + FA × 100)/SOC, and HI (dimensionless) by [TEC − (HA + FA)]/(HA + FA).

To measure soil carbon mineralization, 25 g of sample were placed in closed glass jars and incubated in the dark at field capacity and at 30 °C. The CO_2_ evolved was trapped by 0.5 N NaOH daily for the first week of incubation, every two days for the next two weeks, and determined by titration of the excess NaOH with 0.5 N HCl [33]. 

The soil’s physical and mechanical parameters were characterized according to the official methods of the Ministry of Agriculture (Italy) [34]: monoaxial compression test on soil [35] was detected with an odometer apparatus (controls, mod. T302) under vertical loads (10 and 200 daN) for evaluating the plastic soil deformation during a 24-h test; soil shear strength was measured by using a vane shear test meter (controls, torsion wrench 0–32 nm, diameter 25 mm, vane height 50 mm). For each plot, 20 measures were taken on topsoil (at depths of 0.35–0.70 m); shear resistance (kPa) was calculated with the Terzaghi and Peck [36] relationship to applied torque. Soil penetration resistance was measured on each treatment and on the control areas using a hand penetrometer (controls, Italy) with a 60° cone and base area of 100 mm^2^; maximum hydraulic capacity was measured using the Richards’ method [37].

### 4.3. Soil Total Heavy Metal and DTPA Extraction

Soil total heavy metals (Cu, Zn, Ni, and Pb) were extracted by wet digestion with a HNO_3_-HClO_4_ mixture (2.5:1 ratio) at 140 °C for 40 h. The DTPA-extractable heavy metals were determined according to the Lindsay and Norvell procedure [38]. This method is utilized to estimate the amount of soil heavy metals bioavailable to plant roots in both natural and cropped, neutral or alkaline soils [39] Trace elements in the extracts were analyzed by inductively coupled plasma emission spectroscopy (ICP).

### 4.4. Statistical Analysis

Mean values were compared with the least significance differences testing (LSD) with a confidence level of 95% to test significance (*p* ≤ 0.05). Values marked with the same letter do not differ significantly at *p* ≤ 0.05 (LSD test), *n* = 12.

## Figures and Tables

**Table 1:** Concentration of SOC, TEC and (HA + FA) in soil (g kg^−1^ d.w.).

Treatment	SOC	TEC	(HA + FA)
T	8.6 a	5.1 a	3.3 a
L	10.8 b	6.0 ab	4.1 b
D	11.0 b	7.1 b	4.2 b
C	10.8 b	8.0 bc	5.1 c

Within each column, values marked with the same letter do not differ significantly at *p* ≤ 0.05 (LSD test), *n* = 12.

**Table 2:** Soil physical and mechanical parameters after eight years of sludge administration

Treatment	Soil Deformation at 10 daN (%)	Soil Deformation at 200 daN (%)	Shear Strength (kPa)	Cone Penetration Resistance at 100 mm Depth (MPa)	Maximum Hydraulic Capacity (%)
T	24.13 b	38.8 b	19.65 b	2.89 b	28.72 a
L	27.42 b	39.12 b	20.32 b	3.14 b	27.43 a
D	25.18 b	37.11 b	20.14 b	2.77 b	29.15 a
C	18.33 a	27.42 a	15.54 a	1.81 a	34.68 b

Within each column, values marked with the same letter do not differ significantly at *p* ≤ 0.05 (LSD test), *n* = 12.

**Table 3:** Total concentrations of Cu, Pb, Ni, and Zi in soil (mg kg ^−1^ d.w.)

Treatment	Cu	Pb	Ni	Zn
T	79.0 a	18.50 a	38.42 a	95.25 a
L	89.63 a	18.37 a	42.0 b	98.0 a
D	89.19 a	20.89 a	41.21 b	120.63 b
C	87.13 a	19.84 a	41.28 b	113.0 b

Within each column, values marked with the same letter do not differ significantly at *p* ≤ 0.05 (LSD test), *n* = 12.

**Table 4:** Bioavailable concentrations of Cu, Pb, Ni, and Zi in soil (mg kg ^−1^ d.w.)

Treatment	Cu-DTPA	Pb-DTPA	Ni-DTPA	Zn-DTPA
T	14.29 a	2.36 b	0.44 a	2.52 a
L	16.71 ab	1.84 a	0.79 b	5.22 bc
D	18.49 b	2.68 b	1.08 c	6.82 c
C	17.33 b	2.24 b	0.90 b	4.87 b

Within each column, values marked with the same letter do not differ significantly at *p* ≤ 0.05 (LSD test), *n* = 12.

**Table 5:** Characteristics of the sewage sludge (average values) and limits (D.L. 27 January 1992 n. 99). Values refer to dry weight.

Parameter	Liquid Sludge (L)	Dehydrated Sludge (D)	Composted Sludge (C)	Limits
Dry matter (g kg^−1^)	29.91	243	622	
pH	8	8	7	≥5.5
Organic C (g kg^−1^)	319	286	222	≥200
Total Cu (g kg^−1^)	906	934	666	≤1000
Total Ni (g kg^−1^)	202	221	161	≤300
Total Pb (g kg^−1^)	125	118	109	≤750
Total Zn (g kg^−1^)	1514	1574	1140	≤2500

**Table 6:** Physical and chemical characteristics of soil at the beginning of the study before the sludge amendment and the limits defined by Italian Law for the use of sewage sludge on agricultural soil (D.L. 27 January 1992 n. 99). Concentrations refer to dry weight.

Parameter	Value	Limits	Parameter (mg kg^−1^)	Value	Limits
Sand %	23		Available P	16	
Silt %	55		Total Cu	68.2	≤100
Clay %	22		Total Ni	43.8	≤75
Texture (USDA)	Silt loam		Total Pb	14.9	≤100
pH	7.8	7.5 > pH > 6	Total Zn	78.5	≤300
SOC (g kg^−1^)	8.6				
OM (g kg^−1^)	14.83				
Total N (mg kg^−1^)	1180				
C/N	7.6				
C.E.C. (cmol kg^−1^)	13.8	>15			
